# Translating cardiorheumatology into practice: a multilevel pilot network

**DOI:** 10.1093/ehjcvp/pvag070

**Published:** 2026-09-11

**Authors:** Gian Luca Erre, Fabio Cacciapaglia, Giuseppe Damiano Sanna, Fabiola Atzeni, Elena Bartoloni, Crescenzio Bentivenga, Claudio Borghi, Elisa Gremese, Andreina Manfredi, Matteo Piga, Garifallia Sakellariou, Marinica Savino, Ombretta Viapiana, Francesca Romana Spinelli

**Affiliations:** Dipartimento di Medicina, Chirurgia e Farmacia, Università degli Studi di Sassari, Sassari, Italy; SSD Reumatologia, Azienda Ospedaliero-Universitaria di Sassari, Sassari, Italy; Dipartimento di Medicina e Chirurgia, Università LUM Giuseppe Degennaro, Casamassima-Bari, Italy; SC Cardiologia Clinica e Interventistica, Azienda Ospedaliero-Universitaria di Sassari, Sassari, Italy; Dipartimento di Medicina Clinica e Sperimentale, Università degli Studi di Messina, Messina, Italy; Dipartimento di Medicina e Chirurgia, Università degli Studi di Perugia, Perugia, Italy; Medicina Interna cardiovascolare, Policlinico di Sant'Orsola, Bologna, Italy; Dipartimento di Scienze Mediche e Chirurgiche, Università Alma Mater, Bologna, Italy; Rheumatology and Clinical Immunology, IRCCS Humanitas Research Hospital, Rozzano-Milano, Italy; Reumatologia, Azienda Ospedaliera Universitaria Policlinico di Modena, Modena, Italy; Department of Medical Sciences and Public Health, Universitá degli Studi di Cagliari, Italy; SC Reumatologia, Azienda Ospedaliero-Universitaria di Cagliari, Monserrato-Cagliari, Italy; Department of Internal Medicine and Therapeutic, Università degli Studi di Pavia, Pavia, Italy; Istituti Clinici Scientifici Maugeri IRCSS, Pavia, Italy; DipartimentoScienze Cardiovascolari - CUORE, Fondazione Policlino Universitario Agostino Gemelli IRCCS-Università Cattolica del Sacro Cuore, Roma, Italy; Medicina, UNiversità degli Studi di Verona, Verona, Italy; Scienze Mediche e Cardiovascolari, Sapienza University of Rome, Roma, Italy

To the Editor,

We read with interest the article by Semb *et al*.^1^ on the scientific rationale for establishing cardiorheumatology as a dedicated field. While defining this subspecialty is needed, the next critical step is to move from conceptual advocacy to practical application.

Currently, international models of cardiorheumatology exhibit varying degrees of integration between clinical research and routine care. The UK CARDIO-IMID (cardiovascular involvement in immune-mediated inflammatory diseases) partnership uses public and charitable funding to sustain a multicentre research infrastructure across more than 30 sites^2^; in contrast, North American initiatives largely operate as single-centre academic hubs within public or private nonprofit systems. While research networks foster cross-specialty collaboration, physical, standalone cardiorheumatology clinics are unlikely to be feasible at scale. A more pragmatic approach, in our opinion, is a multi-level network –analogous to structured cardio-oncology services^3^—that stratifies care according to clinical complexity, optimizing existing resources and structured collaboration (*Figure 1*).

**Figure 1:**
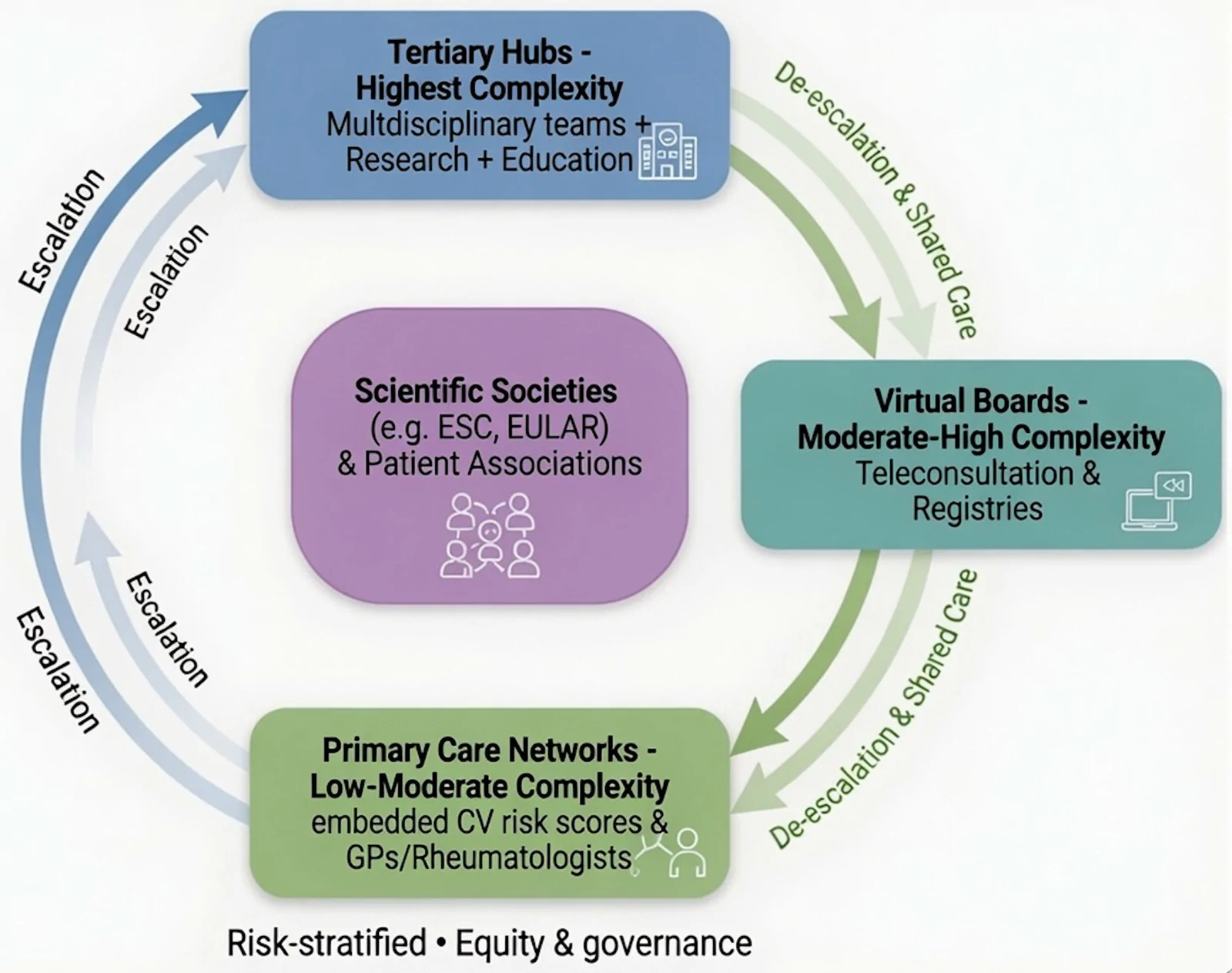
Proposed multilevel pilot cardiorheumatology network. The diagram illustrates the flows between different levels of care complexity of the cardiorheumatology network pilot programme with the central role of scientific societies and patient associations.

At the highest level of complexity, multidisciplinary teams should be established within existing academic tertiary centres. These teams would manage high-risk patients with severe conditions often requiring advanced diagnostic and interventional procedures. They are uniquely positioned to advance translational research into the drivers of vascular and myocardial disease in rheumatic diseases, generating trial and real-world data on novel risk models^4,5^ and cross-specialty therapies (e.g. glucagon-like peptide 1 receptor agonists, Interleukin-1 beta inhibitors, Interleukin-6 inhibitors, colchicine)^6–8^. Demonstrating the clinical utility of these agents may facilitate controlled, earlier access within existing local regulatory and budgetary boundaries. In this regard, dedicated registries are essential to monitor real-world safety and long-term outcomes, tracking both potential toxicities and additional clinical effects. In parallel, these teams should collaborate with both cardiology and rheumatology scientific societies to develop joint consensus guidelines and implement educational programmes for resident physicians, community specialists, and general practitioners. Exemplifying this strategy, the Italian Society for Rheumatology’s CORDIS (Cardiovascular, Obesity, and Rheumatic Disease) study group actively integrates cross-specialty research, consensus recommendations, and physician training.^9^

Complex subacute cases can be evaluated by virtual multidisciplinary boards (MDT)—modelled on molecular tumour boards,^10^ extending specialist expertise to peripheral centres via existing telemedicine platforms linked to cross-disciplinary registries. At the community level, primary care physicians alongside outpatient cardiologists and rheumatologists act as primary gatekeepers while driving cardiovascular risk prevention and routine monitoring to achieve comprehensive disease control of the underlying rheumatic condition.

To optimize access to tertiary care, referral algorithms modelled on established cardio-oncology frameworks^11^ should be adopted. Implementation requires a standardized electronic form jointly developed by cardiology and rheumatology societies and approved by tertiary hubs and primary care structures. Triage can be based on specific cardiac biomarkers and clinical scenarios, stratifying patients into urgent referrals and subacute presentations. While acute clinical conditions are directed immediately to tertiary emergency departments of the cardiorheumatology network, complex subacute cases can be evaluated by virtual MDT. Finally, closing the care loop requires formal interlevel agreements to govern step-down back-transfer, transitioning stabilized patients to intermediate monitoring settings before return to primary and community care—ensuring a resource-efficient continuum of care.

Logically, the success of the proposed entire network depends on primary prevention. To this end, a pragmatic operative model based on risk stratification relying on accessible tools should be implemented in primary and community care. Such a risk evaluation system, adapting structured cardio-oncology templates,^3^ should ideally integrate baseline algorithms (e.g. SCORE2), disease-specific risk modifiers (reflecting different autoimmune phenotypes and current disease activity), and concurrent exposure to antirheumatic therapies with intrinsic cardiometabolic risks (e.g. glucocorticoids). Implementing this risk-tailored strategy is essential to achieving guideline-recommended targets and overcoming therapeutic inertia.

Government authorities, scientific societies, and patient organizations should also collaborate to implement awareness campaigns that highlight the dual contributions of traditional factors and ongoing disease activity to cardiovascular risk, guaranteeing engagement and therapeutic adherence in a population already burdened by chronic pain and complex polypharmacy.

However, several structural barriers hinder immediate large-scale implementation of this cardiorheumatological network, ranging from rigid spending limits in public single-payer systems, to incomplete system interoperability and missing reimbursement codes in commercial markets, and digital infrastructure deficits in resource-limited settings.

To establish governance and demonstrate feasibility, this framework should be piloted within a defined administrative area—such as a single provincial health district in Italy—and anchored by an academic tertiary hub with expertise in the management of complex cardiorheumatology patients. Initial financing can be supported by competitive research grants, local innovation grants, or public–private research partnerships, bypassing initial systemic budgetary constraints. Crucially, rigorous evaluation of this pilot programme requires a prespecified matrix of key performance indicators encompassing workflow efficiency (e.g. time-to-MDT resolution, appropriate referral rate), clinical efficacy (e.g. comprehensive cardiovascular risk stratification rate, rheumatologic target attainment rate, cardiovascular guidelines adherence rate, major adverse cardiovascular events risk reduction, patient-reported outcome measure response rate), and health-economic value (e.g. incremental cost-effectiveness ratio). Only by demonstrating clinical benefit, cost-effectiveness, sustainability, and scalability, a pilot programme could pave the way for transitioning cardiorheumatology from a theoretical concept into a concrete healthcare model.

Sincerely,

Erre GL, Cacciapaglia F, Sanna GD, Atzeni F, Bartoloni Bocci E, Bentivenga C, Borghi C, Gremese E, Manfredi A, Piga M, Sakellariou G, Savino M, Viapiana O, Spinelli F.R.

## Data Availability

The data underlying this article are available in the article and in its online supplementary material.
